# Natural Compound Shikonin Is a Novel PAK1 Inhibitor and Enhances Efficacy of Chemotherapy against Pancreatic Cancer Cells

**DOI:** 10.3390/molecules27092747

**Published:** 2022-04-24

**Authors:** Wenjing Ji, Xiaoyan Sun, Yang Gao, Man Lu, Lingxia Zhu, Dawei Wang, Chunping Hu, Jiao Chen, Peng Cao

**Affiliations:** 1 School of Medicine & Holistic Integrative Medicine, Nanjing University of Chinese Medicine, Nanjing 210023, China; 18852675600@163.com; 2 Affiliated Hospital of Integrated Traditional Chinese and Western Medicine, Nanjing University of Chinese Medicine, Nanjing 210028, China; xiaoyande126@126.com (X.S.); gaoyang12022@163.com (Y.G.); lu17513677621@163.com (M.L.); zhulingxia0419@163.com (L.Z.); cleverwdw@126.com (D.W.); njhcp66@126.com (C.H.)

**Keywords:** p21-activated kinase 1, pancreatic cancer, shikonin, apoptosis, synergistic effect

## Abstract

Shikonin is the main component of root extracts from the Chinese herbal medicine *Lithospermum erythrorhizon*, which is commonly used for the treatment of various diseases including cancer. Previous research showed that shikonin suppressed pancreatic cancer growth; nevertheless, its molecular targets and mechanisms have not been elucidated. This study aimed to investigate the interaction and regulatory mechanisms of shikonin on its potential target p21-activated kinase 1 (PAK1). Through a labchip-based screening method, shikonin was identified as a potential bioactive PAK1 inhibitor. Molecular docking technology was used to detect the interaction sites of shikonin and PAK1 kinase. Western blot was performed to validate the mechanism. MTT and flow cytometry were practiced to investigate the effect of shikonin against pancreatic cancer cells. The results show that shikonin significantly inhibited the activity of PAK1 kinase with IC_50_ value of 7.252 ± 0.054 μM. Molecular docking studies showed that shikonin binds to the ATP-binding pocket of the PAK1 kinase domain. Moreover, shikonin inhibited PAK1 activation and its downstream signaling pathway proteins, while reducing proliferation and inducing apoptosis of pancreatic cancer cells. Further studies showed that the treatment of shikonin sensitized pancreatic cancer cells to chemotherapeutic drugs. These results suggest that shikonin, a potential natural inhibitor targeting PAK1 kinase, has promising potent applications in the treatment of pancreatic cancer and chemotherapy sensitization.

## 1. Introduction

Pancreatic cancer is a highly lethal malignancy with poor prognosis and an overall five-year survival rate less than ten percentage [1]. Complete surgical intervention is the only potentially curative treatment for pancreatic cancer [2]. However, most patients are diagnosed at an advanced stage, and may be ineligible for surgical resection because of distant metastases or major vessel involvement [3]. To date, gemcitabine (Gem)-based chemotherapy still plays an important role in the treatment of pancreatic cancer and as an adjuvant therapy after surgical resection [4]. Although a large number of clinical trials with different combinations of chemotherapy have been conducted to identify reasonable regimens that can prolong the survival time of patients, the side effects are numerous, with only modest improvements in survival [5,6]. Therefore, there is an urgent need to explore new therapeutic approaches and drug combinations for patients with pancreatic cancer.

The p21-activated kinase (PAK) family is a class of non-receptor serine/threonine protein kinases that are typically activated by the small-molecule GTPase RAC1 and cell division cycle 42 (Cdc42). PAKs regulate multiple biological events and are located at the intersection of multiple signaling pathways required for tumorigenesis. The PAK family of proteins has six members and can be classified into Class I PAKs (including PAK1–3) and class II PAKs (including PAK4–6) based on their structural and functional similarities [7]. Increasing evidence shows that PAK1 is frequently amplified or overexpressed in patients with a variety of malignant tumors, such as ovarian, hepatocellular carcinoma, breast, and colorectal cancers [8,9,10,11]. Furthermore, our previous studies and those of others have shown that PAK1 is overexpressed in most pancreatic cancer tissue samples and specifically inhibiting or knockdown PAK1 reduced cell growth both in vitro and in vivo [12,13]. Besides, PAK1 inhibitors also significantly suppressed tumor invasion and metastasis of in situ xenograft models of human pancreatic cancer cell lines with GEM resistance [14]. Therefore, PAK1 is expected to be a potential target for pancreatic cancer treatment and chemotherapy sensitization, and the screening and development of specific inhibitors are of great significance.

Natural products are the most important sources of lead compounds for drug discovery and composed some of the most widely used anticancer drugs. Shikonin is the major active ingredient isolated from Zicao (*Lithospermum erythrorhizon* or *Arnebia euchroma* (*Royle*) *Johnst*), which is a Chinese medicinal herb with the effect of promoting blood circulation and detoxifying, etcs. It has been extensively used in clinical for centuries to treat several kinds of diseases, including viral infection, burn, inflammation and cancer [15]. Over the past decades, shikonin has been shown as anticancer activity via multiple kinds of mechanisms. For example, shikonin induces apoptosis and autophagy of colorectal cancer cells by targeting the galectin-1/JNK signaling axis [16]. Shikonin inhibits proliferation and promotes apoptosis of gastric cancer cells through the PI3K/Akt signaling pathway [17]. Moreover, shikonin has been shown to trigger ROS-based mitochondria-mediated apoptosis and significantly inhibited tumor growth in a human colon cancer SW480 xenograft mouse model [18]. Recently, shikonin has been reported to mediate PD-L1 degradation by inhibiting NF-κB/STAT3 and NF-κB/CSN5 signaling pathways and blocking immune evasion in pancreatic cancer [19].

However, the question of whether shikonin executes anti-pancreatic cancer activity through PAK1 has not been fully revealed yet. In this work, we demonstrated that shikonin significantly inhibited PAK1 activity and its downstream signaling pathways by binding to the ATP pocket. Furthermore, we found that shikonin decreased the proliferation of pancreatic cancer cells and exhibited a synergistic effect with chemotherapeutic drugs. These data suggest that shikonin, an inhibitor of PAK1 kinase, has potential applications in pancreatic cancer therapy.

## 2. Materials and Methods

### 2.1. Reagents

The TargetMol_Natural_Compound_Library was provided by the Core Technology Facility of Center for Excellence in Molecular Cell Science, CAS. Shikonin, baicalein, sanguinarine, proflavine hemisulfate, and celastrol were purchased from Herbest, Ltd. (Shaanxi, China). The purity of these compounds was over 98%, which was determined by high-performance liquid chromatography. Primary antibodies against p-PAK1 (Lot #2606), PAK1 (Lot #2602), p-cRAF (Lot #9427), c-RAF (Lot. #9422), p-MEK1(Lot. #9127), MEK1(Lot. #2352), p-mTOR (Lot. #5530), and mTOR (Lot. #2983) were purchased from Cell Signaling Technology (Danvers, MA, USA). GAPDH (Lot. # AA66122) antibody was from Bioworld Technology, Inc. (Suite 500 St. Louis Park, MN, USA). Reagents for cell culture were purchased from Gibco (Grand Island, NY, USA).

### 2.2. Cell Lines

Human pancreatic cancer cell lines (BxPC-3 and PANC-1) were purchased from the Cell Bank of Shanghai Institute of Biochemistry and Cell Biology (Shanghai, China). Cells were cultured in Dulbecco’s Modified Eagle Medium (DMEM) supplemented with 10% fetal bovine serum (FBS) and 1% penicillin/streptomycin.

### 2.3. Determination of Enzymatic Activity Inhibition

Human recombinant PAK1 (BPS Bioscience Inc., San Diego, CA, USA) at 0.75 μM was premixed with 0.15 μM synthetic substrate Ser/Thr 19 peptide (Sequence KKRNRRLSVA) for 15 min and then incubated with various concentrations of compound in kinase assay buffer (40 mM Tris, 20 mM MgCl_2_ and 0.1 mg/mL BSA) at pH 7.5. ATP was then added to initiate the reaction with a total volume of 10 μL, which was incubated at 37 °C for 30 min. The PAK1 level was detected using the ADP-Glo^TM^ kinase assay kit (Promega Corp., Madison, WI, USA) according to the manufacturer’s instructions. The inhibition rate of the compound on PAK1 was calculated using GraphPad Prism8.0 software (GraphPad Co., San Diego, CA, USA).

### 2.4. Molecular Docking

The complex structure, 4EQC (PDB ID), of PAK1 binding to competitive inhibitors was downloaded from the Protein Data Bank (PDB) database (http://www.rcsb.org (accessed on 20 December 2021)) [20]. Protein structures were preprocessed using the Prep Wiz module of Schrodinger 2015-3 software. In the Glide module, the original ligand site of the crystal structure was used as the center, and the surrounding 15 Å range was defined as the active pocket (ATP-binding pocket) to generate a grid file for subsequent molecular docking. In the LigPrep module, small molecules are protonated and hydrogenated, and energy is minimized using the OPLS3 force field to obtain the optimal conformation. Molecular docking of the prepared receptors and ligands was performed in Glide, and the docking mode was standard precision (SP).

### 2.5. Assessment of Cell Proliferation and Apoptosis

BxPC-3 and PANC-1 cells were seeded in 96-well plates at a density of 5 × 10^3^ cells/well. After 24 h, the cells were treated with the compounds at serial dilutions ranging from 0 to 40 μM. Cell viability was determined using the 3-(4,5-dimethylthiazol-2-yl)-2,5-diphenyltetrazolium bromide (MTT) or cell counting kit (CCK)-8 assays, as described previously [21]. The externalization of phosphatidylserine was analyzed using an Annexin V/7-AAD apoptosis detection kit (BD Biosciences, Franklin Lakes, NJ, USA), according to the manufacturer’s instructions.

### 2.6. Western Blot Analysis

BxPC-3 and PANC-1 cells were treated with 0, 0.5, 1, 1.5, 2, 3 μM shikonin for 24 h, or 3 μM shikonin for 0, 3, 6, 9, 12, 24 h, and lysed in RIPA buffer containing protease and phosphatase inhibitors. The proteins were separated by SDS-PAGE and then transferred to PVDF membranes. After being blocked with 5% skim milk, the membranes were incubated with the indicated primary antibodies at 4 °C overnight, then washed and incubated with secondary antibodies conjugated with IgG DyLight at room temperature for 1 h. Finally, the membranes were washed and scanned using an Odyssey infrared fluorescence scanner (LI-COR Biosciences, Lincoln, NE, USA).

### 2.7. Combination Index (CI)

The combined effects of shikonin and chemotherapeutic drugs (Gem and 5-fluorouracil [5-FU]) were quantified through the Chou–Talalay method [22,23]. The inhibition values for cells treated with shikonin alone, chemotherapeutic drugs alone, or shikonin combined with a chemotherapeutic drug were measured. The combination index (CI) was calculated using CompuSyn software (Biosoft, Cambridge, UK), and the values were interpreted as follows: <1.0, synergistic; 1.0, additive; and >1.0, antagonistic [23].

### 2.8. Statistical Analysis

All experiments were repeated at least three times, and the data are presented as the means ± (SD), unless noted otherwise. Statistical analysis of the differences between multiple groups was done by one-way ANOVA. *p* values less than 0.05 were considered to be statistically significant.

## 3. Results

### 3.1. Screening of PAK1 Inhibitors

**Figure 1:**
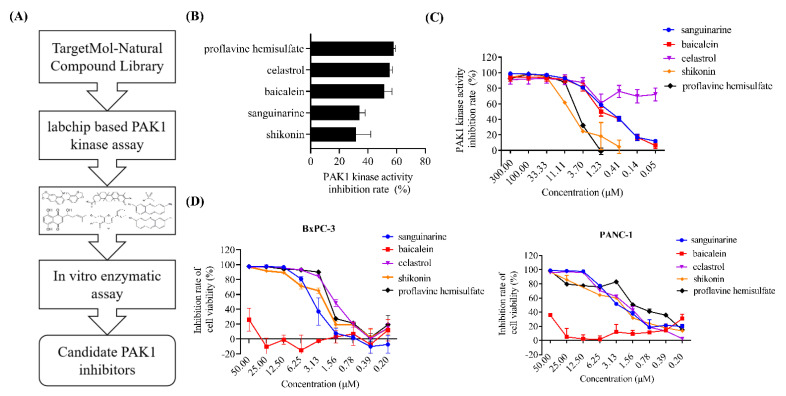
Screening of PAK1 inhibitors. (**A**) Process for screening PAK1 inhibitors from natural compound library. (**B**) Human PAK1 protein was incubated with indicated compounds at 5 μM and analyzed using labchip−based PAK1 assay. (**C**) Human PAK1 protein was incubated with indicated compounds and analyzed using ADP−Glo^TM^ kinase assay kit. (**D**) BxPC−3 and PANC−1 cells were treated with indicated compounds for 48 h and analyzed using MTT assay. Results are means ± SD of three independent experiments.

We previously established a labchip-based screening method for PAK1 inhibitors [24]. In this study, we aimed to screen novel PAK1 inhibitors from natural compounds which have great potential in cancer therapy because of their safety, low cost, and oral bioavailability. The screening process is illustrated in Figure 1A, and the primary screening identified five natural compounds (baicalein, sanguinarine, proflavine hemisulfate, celastrol, and shikonin) with PAK1 inhibitory activity (Figure 1B). Human PAK1 protein was then incubated with the compounds at various concentrations (0.05–300 μM), and the kinase activity was carefully measured.

The results confirmed that all the compounds significantly inhibit the activity of PAK1 with a half maximal inhibitory concentration (IC_50_) ranging from 3.614 ± 0.007 to 7.244 ± 1.99 μM (Figure 1C). Furthermore, we tested their cytotoxicity against pancreatic cancer cells using MTT assay. The compounds except for baicalein exhibited significant inhibitory effects on the proliferation of both BxPC-3 and PANC-1 pancreatic cancer cells (Figure 1D). In BxPC-3 cells, the IC_50_ of sanguinarine, proflavine hemisulfate, celastrol and shikonin were 3.769 ± 0.799 μM, 1.879 ± 0.108 μM, 1.565 ± 0.057 μM and 2.791 ± 0.408 μM, respectively. In PANC-1 cells, the IC_50_ of several compounds were 2.233 ± 0.252 μM, 1.189 ± 0.197 μM, 2.209 ± 0.153 μM and 4.018 ± 0.812 μM, respectively. Among these compounds, shikonin is the main bioactive component of the traditional Chinese medicine formulation “Zicao”, which has a long history of clinical application [25]. Therefore, we chose shikonin for further investigation of its potential for clinical development.

### 3.2. Shikonin Inhibits PAK1 by Interacting with ATP Pocket

**Figure 2:**
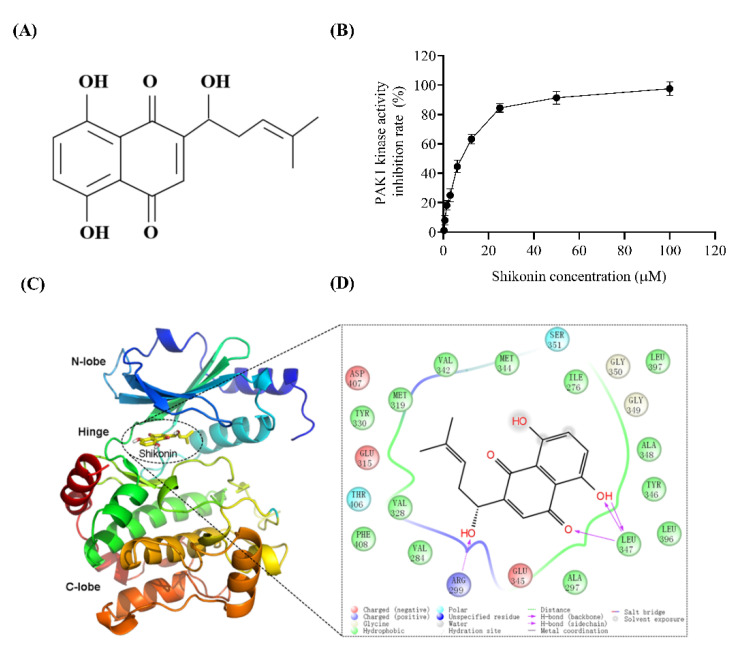
Shikonin binds to ATP pocket of PAK1. (**A**) Structure of shikonin. (**B**) Human PAK1 protein was incubated with shikonin at indicated concentrations and kinase activity was measured. Results are means ± SD of three independent experiments. (**C**) Molecular docking of shikonin and ATP pocket of PAK1. (**D**) Specific binding mode of shikonin and PAK1.

The structure of shikonin is shown in Figure 2A. The assay of serially diluted concentrations of shikonin (0.39–100 μM) against human PAK1 protein showed an IC_50_ of 7.252 ± 0.054 μM in vitro (Figure 2B). To investigate the mechanism underlying the action of shikonin on PAK1, the complex structure of PAK1 combined with a competitive inhibitor (PDB ID: 4EQC) was used for molecular docking. PAK1 was mainly composed of N- and C-lobes, and the middle was connected by a hinge area.

The ATP-binding pocket is located at the interface of the N-lobe and C-lobe, which is also the site of interaction between shikonin and PAK1 (Figure 2C). The specific binding modes of comfrey and PAK1 are shown in the figure below (Figure 2D). The carbonyl and hydroxyl groups on the naphthoquinone ring formed multiple hydrogen bonds with the Leu347 skeleton, and the hydroxyl groups formed hydrogen bonds with the side chain of Arg299. In addition, shikonin also showed hydrophobic interactions with Ile276, Ala297, Val284, Leu396, Met319, Val328, Val342, Met344, Tyr346, and Ala348.

### 3.3. Shikonin Inhibited the Growth of Pancreatic Cancer Cells

**Figure 3:**
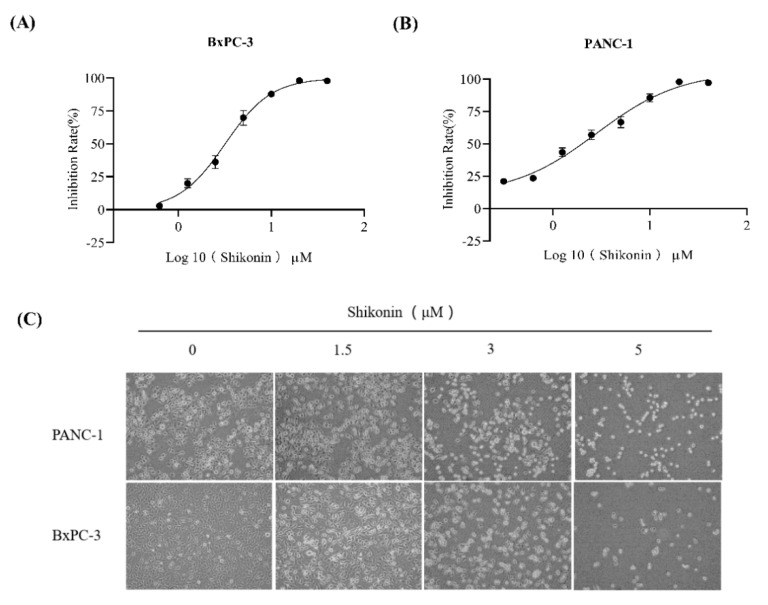
Effects of shikonin on pancreatic cancer cells. (**A**) BxPC−3 and (**B**) PANC−1 cells were treated with shikonin for 24 h and analyzed using MTT assay. (**C**) PANC−1 and BxPC−3 cells were treated with shikonin at 0, 1.5, 3, and 5 μM for 12 h, and images were captured under an Olympus inverted microscope. Results are means ± SD of three independent experiments.

We subsequently explored the cytotoxicity effect of shikonin against pancreatic cancer cells. By MTT assay, we found that shikonin displayed significantly inhibitory effects against the proliferation of BxPC-3 (Figure 3A) and PANC-1 (Figure 3B) cells. The IC_50_ values of shikonin against BxPC-3 and PANC-1 cells were 3.183 ± 0.321 μM and 1.800 ± 0.013 μM, respectively. Furthermore, the inverted microscopy showed that incubation of both BxPC-3 and PANC-1 cells with shikonin for 12 h induced cell rounding and the attachment fell off (Figure 3C).

### 3.4. Shikonin Suppressed Downstream Pathways of PAK1 in Pancreatic Cancer Cells

**Figure 4:**
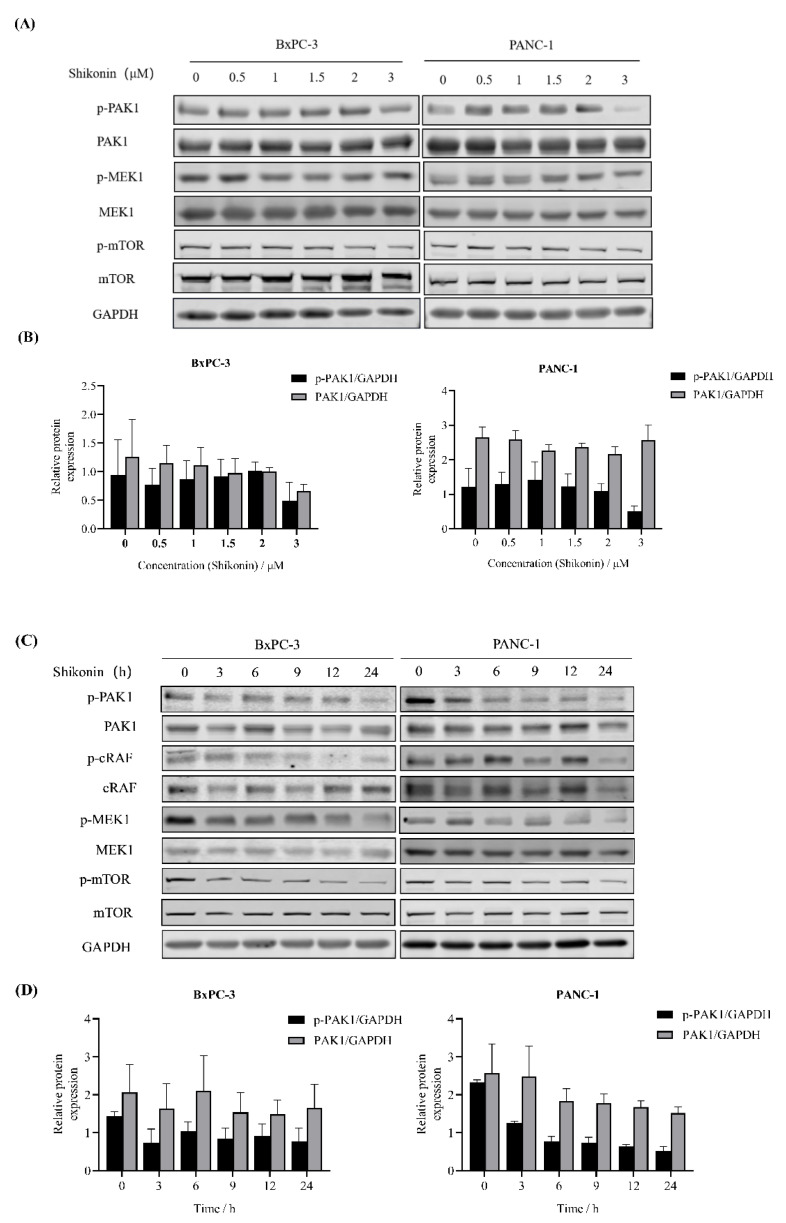
Shikonin inhibits PAK1-related signaling pathways in pancreatic cancer cells. (**A**) BxPC−3 and PANC−1 cells were treated with shikonin for indicated concentrations, and Western blot assay was conducted using indicated antibodies. (**B**) The expression of p−PAK1 and PAK1 was quantified by densitometry analysis and normalized against GAPDH. (**C**) BxPC−3 and PANC−1 cells were treated with shikonin for indicated time points, and Western blot assay was conducted using indicated antibodies. (**D**) The expression of p−PAK1 and PAK1 was quantified by densitometry analysis and normalized against GAPDH. The experiments were conducted at least twice.

PAK1 is involved in the regulation of many important signaling pathways, such as the RAS/ERK, PI3K/AKT and WNT/β-catenin pathways [26,27]. Therefore, we examined whether or not shikonin suppressed the activation of PAK1-related signaling pathways. By Western blot, we showed that the treatment with shikonin at 2 to 3 μM for 24 h strongly decreased PAK1 phosphorylation in PANC-1 cells (Figure 4A,B). Moreover, the inactivation of PAK1 reduced mTOR and MEK1 phosphorylation. In BxPC-3 cells, treatment with shikonin at 2 to 3 μM for 24 h also suppressed the phosphorylation of PAK1 and downstream signaling pathway proteins. These results suggested that shikonin inhibited PAK1-related pathway proteins in a concentration-dependent manner in PANC-1 and BxPC-3 cells. Subsequently, we checked the phosphorylation of PAK1 incubated with shikonin for different time points. In PANC-1 cells, the phosphorylation of PAK1 and downstream proteins (c-RAF, MEK1, and mTOR) decreased after 3 h treatment with shikonin at 3 μM (Figure 4C,D). In BxPC-3 cells, shikonin also depleted the PAK1 signalling pathway in a time-dependent manner.

### 3.5. Shikonin Induced Apoptosis of Pancreatic Cancer Cells

**Figure 5:**
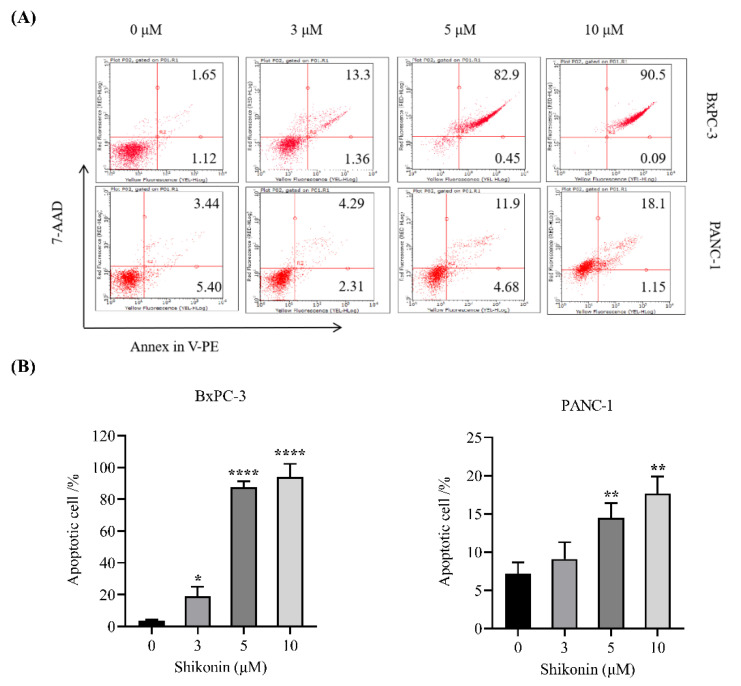
Shikonin induced apoptosis of pancreatic cancer cells. (**A**) BxPC−3 and PANC−1 cells were treated with shikonin for 24 h, and apoptosis was detected using Annexin V/7−AAD staining and flow cytometry. (**B**) Quantification of apoptotic cells. Results are means ± SD of three independent experiments. * *p* < 0.05, ** *p* < 0.01, and **** *p* < 0. 0001.

In order to further explore the activity of shikonin on pancreatic cancer cells, the apoptosis of pancreatic cancer cells was measured by flow cytometry. After 24 h treatment, shikonin at the concentrations of 3 μM, 5 μM and 10 μM caused over 14.66%, 83.35% and 90.50% apoptosis of BxPC-3 cells, respectively (Figure 5A). Similarly, shikonin induced dose-dependent apoptosis of PANC-1 cells. The percentage apoptosis of both cell types was significantly different from that of their respective control groups (Figure 5B).

### 3.6. Shikonin Sensitized Pancreatic Cancer Cells to Chemotherapeutic Drugs

**Figure 6:**
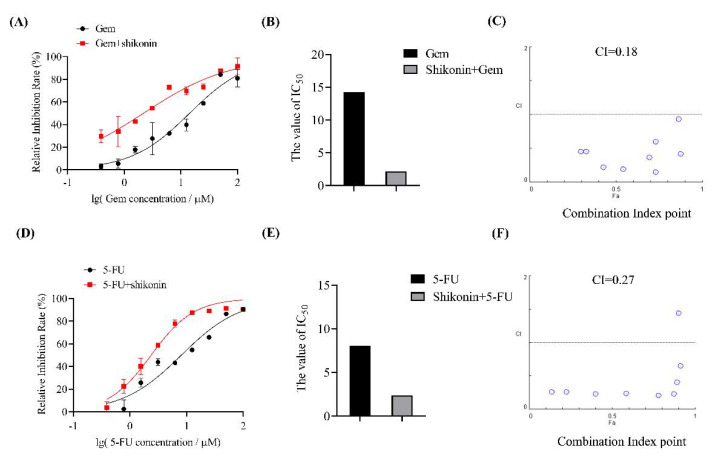
Combination of shikonin and chemotherapeutic agents showed significant synergistic effects against pancreatic cancer cells. (**A**) BxPC−3 cells were treated with Gem with or without shikonin and analyzed using MTT assay. (**B**) IC_50_ values of Gem or Gem plus shikonin in BxPC−3 cells. (**C**) CI of Gem plus shikonin in BxPC−3 cells. (**D**) MTT assay of BxPC−3 cells treated with 5−FU with or without shikonin. (**E**) IC_50_ values of 5−FU with or without shikonin in BxPC−3 cells. (**F**) CI of 5−FU and shikonin in BxPC−3 cells. Results are means ± SD of three independent experiments.

To analyze the activity of shikonin combined with chemotherapeutic drugs, MTT assay was used to investigate the inhibitory effect of combined treatment against pancreatic cancer cells. As shown in Figure 6A, shikonin obviously enhanced the cytotoxicity effect of chemotherapeutic drugs Gem against BxPC-3 cells. Co-treatment with shikonin decreased the IC_50_ of Gem from 14.22 to 2.181 μM (Figure 6B). Further analysis revealed that shikonin showed a strong synergistic effect with Gem on BxPC-3 cells with a CI of 0.18 (Figure 6C). Shikonin also sensitized BxPC-3 cells to the cytotoxicity of 5-FU (Figure 6D). Cotreatment with shikonin decreased the IC_50_ of 5-FU from 8.071 to 2.371 μM (Figure 6E), with a CI as low as 0.27 (Figure 6F). Taken together, the results showed that the combination of shikonin and Gem or 5-FU has a significant synergistic effect on pancreatic cancer cells.

## 4. Discussion

Increasing evidence shows that PAK1 is overexpressed in pancreatic cancer patients, and the deregulation of PAK1 contributes to increased cancer cell survival, proliferation, migration and Gem resistance [28,29,30]. MUC13, a newly discovered transmembrane mucin that was found to be overexpressed in pancreatic cancer, is associated with dysregulation of PAK1 [31]. Downregulated PAK1 in pancreatic cancer cells displayed decreased proliferation and transforming properties, and PAK1 diminished cells could not form xenografts in athymic mice [32]. Previous studies demonstrated that PAK1 inhibitors had a marked antitumor effect in pancreatic murine models. Therefore, PAK1 may serve as a potential therapeutic target for pancreatic cancer. To date, several PAK1-targeted therapeutic compounds have been developed, including ATP-competitive inhibitors (Indolocarbazoles, aminopyrimidines, and Bis-anilino pyrimidines), allosteric inhibitors (Naphthtols and Dibenzodiazepines), and peptide inhibitors [33]. It is a long way until their use in preclinical/clinical trials.

To searching for novel natural PAK1 inhibitors, we conducted high-throughput screening using a natural compound library. The natural naphthoquinone compound shikonin was identified as a novel PAK1 blocker, enriching the mother nucleus structure of PAK1 inhibitors. The in vitro kinase assay and molecular docking demonstrated that shikonin binds to the interface of the N-lobe and C-lobe of the ATP pocket of PAK1.

Although several PAK1 inhibitors such as CP734, FRAX-597, and G-5555 have been synthesized [12,33,34], further studies are required to support their clinical development and use. In this study, shikonin demonstrated a stronger kinase inhibitory effect on PAK1 and cytotoxicity against pancreatic cells than that previously reported for CP734. Furthermore, shikonin has been demonstrated to inhibit cellular phosphorylation of PAK1 and diminished the downstream signaling pathway. Importantly, shikonin caused significant apoptosis of both PANC-1 and BxPC-3 cells. These findings indicate that shikonin possesses potent therapeutic use in pancreatic cancer. However, further investigations with a series of animal experiments are still required for clinical transformation of the present study.

Previous studies have demonstrated that shikonin is a natural naphthoquinone with anticancer effects against a variety of malignancies in vitro and in vivo [34,35,36]. It mainly exerts its antitumor effect by regulating various signaling pathways such as NF-κB, PI3K/AKT/mTOR and MAPKs [37]. However, the exact anti-pancreatic cancer mechanism of shikonin has not been fully elucidated. In the present study, we showed that shikonin targets the ATP pocket of PAK1 to inhibit its activity. Shikonin diminished PAK1 downstream signaling pathways, inhibiting proliferation and inducing apoptosis of pancreatic cancer cells. The combination of shikonin and chemotherapeutic drugs demonstrated strong synergistic effects. The results of this study revealed that PAK1 is a new target of shikonin, and that inhibition of the PAK1 downstream signaling pathways is a mechanism of shikonin-induced cytotoxicity in pancreatic cancer cells.

## 5. Conclusions

In this study, the natural product shikonin was identified as a novel PAK1 inhibitor that significantly inhibited the growth and induced apoptosis of pancreatic cancer cells. Our findings showed that shikonin acts as a potent bioactive inhibitor of PAK1 and is a candidate anticancer natural product with considerable potential value that warrants further investigation.

## Data Availability

The data in this study are available on request from the corresponding author.

## Sample Availability

Compounds used in this study are commercially available.
